# Registration of 3D and Multispectral Data for the Study of Cultural Heritage Surfaces

**DOI:** 10.3390/s130101004

**Published:** 2013-01-15

**Authors:** Camille Simon Chane, Rainer Schütze, Frank Boochs, Franck S. Marzani

**Affiliations:** 1 i3mainz, Fachhochschule Mainz, Lucy-Hillebrand-Straße 2, 55128 Mainz, Germany; E-Mails: camille.simon@u-bourgogne.fr (C.S.C.); rainer.schuetze@geoinform.fh-mainz.de (R.S.); frank.boochs@geoinform.fh-mainz.de (F.B.); 2 le2i, Université de Bourgogne, B.P. 47870, 21078 Dijon CEDEX, France

**Keywords:** 2D-3D registration, close range photogrammetry, optical calibration, 3D digitization, multispectral imaging, cultural heritage

## Abstract

We present a technique for the multi-sensor registration of featureless datasets based on the photogrammetric tracking of the acquisition systems in use. This method is developed for the in situ study of cultural heritage objects and is tested by digitizing a small canvas successively with a 3D digitization system and a multispectral camera while simultaneously tracking the acquisition systems with four cameras and using a cubic target frame with a side length of 500 mm. The achieved tracking accuracy is better than 0.03 mm spatially and 0.150 mrad angularly. This allows us to seamlessly register the 3D acquisitions and to project the multispectral acquisitions on the 3D model.

## 1. Introduction

3D digitization and multispectral analysis are both increasingly used for the study of cultural heritage. 3D models provide an accurate representation of the shape of the object under study. It is easy for conservators and non-specialists to share these models and interact with them without damaging the object. Such models are also used for education and communication in virtual reality applications.

Multispectral images, on the other hand, provide precise information about the surface reflectance properties of an object. With accurate photometric calibration procedures it is possible to extract data that are independent from both the acquisition system in use and the illumination conditions. This contactless analysis technique can be a step in non-invasive pigment identification [1].

Because of the complementary nature of the data acquired by these two techniques, conservators benefit from augmented 3D models with multispectral texture. The annotation of 3D models and the integration of complementary techniques is widely used for the study of cultural heritage [2–5]. There have been some attempts at creating integrated 3D/multispectral acquisition systems [6–10] for the study of cultural heritage objects. However, such integrated systems lack the flexibility necessary to study a variety of cultural heritage objects. Using separate systems for the 3D and multispectral (2D) acquisitions enables us to independently choose the most suitable for the given application. The drawback is that we then have to register these multiple datasets.

The traditional approach is to use homologous points in the 2D and 3D data to retrieve the unknown intrinsic and extrinsic camera parameters, for example using the Tsai camera calibration method [11–13]. The main defect of this technique resides in the difficulty to identify corresponding points between the 2D and 3D data, be it manually or automatically. Color discrepancies do not necessarily correspond to structural discrepancies and vice versa. Targets may be used to guide the registration process, but they are usually not adapted to cultural heritage applications where we want to minimize the disturbance to the object. Depending on the target resolution and the aimed registration accuracy, many targets may be necessary, partially occluding the object.

The need to find corresponding points is altogether eliminated when the registration of 2D on 3D is based on fully automated maximization of mutual information methods [14–18]. Mutual information is a statistical measure of similarity between two images, which is used to compare the 2D data to be mapped with a rendering of the 3D model. Many rendering methods have been used, including depth maps [16], gradient maps [17], silhouette maps, reflection maps and other illumination-based renderings [18]. The camera parameters are iteratively optimized and a new rendering is created until the registration is achieved. The precision of the ensuing registration is of the order of a few pixels, which is sufficient for visualization purposes.

There is no need to estimate the camera parameters from the data if they are known through calibration and tracking. In theory, magnetic tracking can be used to derive the position and orientation of the sensor in use [19,20]. However, even recent sensors [21] are not sufficiently precise to be the sole registration input. Furthermore, surrounding metals in the acquisition space increase this error to the point of rendering the measures useless [22]. For digitizations performed in a laboratory, a robot can be used to calculate the next best view and to perform the registration based on those known positions and orientations of the acquisition system [23].

Optical tracking is increasingly used for 3D mesh registration. A new generation of handheld laser scanners registers the flow of acquisitions by relying on either a laser tracker [24] or a photogrammetric setup [25–27] to determine the position and orientation of the sensor at each moment. An example of optical tracking for 3D registration in the context of cultural heritage study is given by Blais *et al.* [28]. A painting was scanned with both a high resolution color laser scanner and a lower-resolution laser scanner. The lower-resolution scanner acquired the full painting in a single scan and was also used to project optical markers on the surface of the painting, defining the sub-areas to scan with the high-resolution scanner. White spheres were mounted on the high resolution scanner and the third task of the low resolution scanner was to track the position and orientation of this second scanner while in use.

We extend this type of setup for the multisensor registration of featureless datasets. Our technique relies on close range photogrammetry to track 3D and multispectral acquisition systems. Photogrammetry is the science of measuring the position and shape of objects using photography. In stable and well calibrated setups off-line photogrammetry systems can potentially achieve a measurement precision of up to 1:500,000 with respect to the largest object dimension [29]. To achieve such precision, the intrinsic camera parameters must be determined with great accuracy. These intrinsic parameters are also called the camera interior orientation (I.O.) and include sensor resolution, focal length, lens distortion, principle point offset, pixel ratio, pixel skew, etc. The exterior orientation (E.O.) of a sensor is its position and orientation in a given system. These parameters, as well as the object coordinates, are estimated by recognizing corresponding points in the images.

Our goal is to develop a method that permits the registration of featureless 3D models and multispectral acquisitions, for the study of cultural heritage objects. Simulations have shown that it is possible to track our acquisition systems with an accuracy of 0.014 mm spatially and 0.100 mrad angularly [30]. This paper presents the experimental results performed in a laboratory environment to test the feasibility and the accuracy of the technique. We first describe the method and present the materials used. We then describe the acquisition configuration. The results are presented in several subsections: first the accuracy of the individual calibrations is given. We then look into the tracking accuracy. This is followed by an evaluation of the 3D registration accuracy. We then examine the registration of 3D and multispectral data. The article ends with a conclusion, which presents the limitations and advantages of the technique, as well as a few perspectives.

## 2. Materials and Methods

### 2.1. Method Description

**Figure 1.**
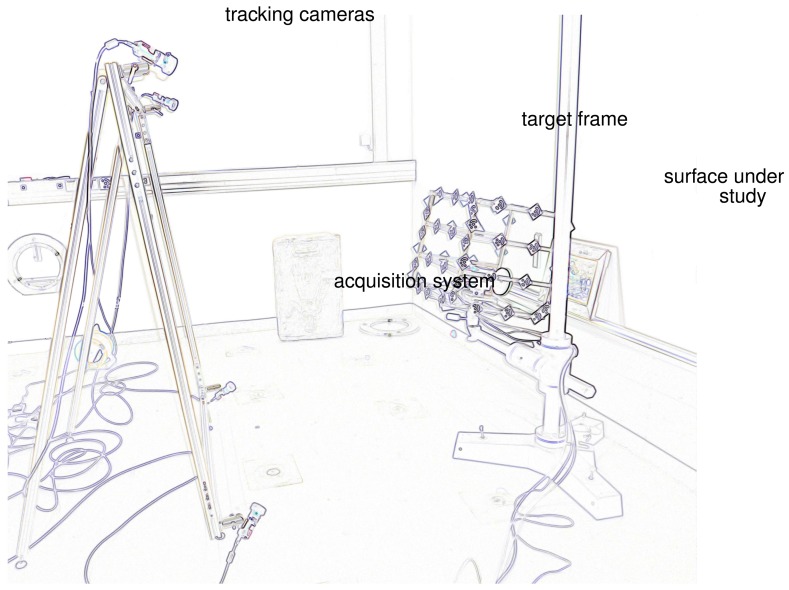
Acquisition overview. An edge detection was performed to improve image readability.

Figure 1 represents the in situ acquisition setup: a group of cameras observe the acquisition systems as they successively digitize the surface under study from various positions. We refer to these as the “tracking cameras” to distinguish them from the multispectral cameras used throughout. The acquisition system is fixed to a target frame that enlarges it and increases the tracking accuracy. The acquisitions are performed simultaneously from the acquisition system in use and the tracking cameras. The registration is performed by projecting the acquired datasets in a single system. To ensure a precise tracking and subsequent registration, all optics and objects in play must be carefully calibrated. We introduce the following coordinate systems, linked to the materials in use:

- *C_Si_*, (*O_Si_*, *x⃗_Si_*, *y⃗_Si_*, *z⃗_Si_*) is the coordinate system linked to acquisition system *i*.
- *C_F_*, (*O_F_*, *x⃗_F_*, *y⃗_F_*, *z⃗_F_*) is the coordinate system linked to the target frame.
- *C_Cj_*, (*O_Cj_*, *x⃗_Cj_*, *y⃗_Cj_*, *z⃗_Cj_*) is the coordinate system linked to each tracking camera *j*. *O_Cj_* is the optical center of the camera, (*x⃗_Cj_, y⃗_Cj_*) define the image plane, *z⃗_Cj_* is collinear to the optical axis.
- *C*_0_, (*O*_0_, *x⃗*_0_, *y⃗*_0_, *Z⃗*_0_) is the world coordinate system.

Additionally, we use the following notations: *A*∣*_C_U__* are the homogeneous coordinates (*x_a_, y_a_, z_a_,* 1) of point *A* in coordinate system *C_U_* and *T_C_V__,_C_U__* is the transformation matrix between two coordinate systems *C_U_* and *C_V_* such that *A*∣*_C_V__* = *T_C_V__,_C_U__* · *A*∣*_C_U__*.

#### Calibration of the target frame

We evaluate the position of each target in *C_F_* using close range photogrammetry by taking many pictures of the target frame surrounded by a few scale bars and additional targets.

#### Calibration of the tracking cameras

It is necessary to know the interior orientation of the tracking cameras. This calibration can be done once the focus of the cameras has been fixed by taking a series of images of a calibration plate in various positions and orientations. The camera calibration is quite stable and can be performed up to a week in advance if we ensure the camera focus is fixed.

#### Calibration of the acquisition system

A similar procedure or a material-specific calibration must be performed to measure the intrinsic parameters of the acquisition systems.

#### Calibration between the target frame and the acquisition system

It is necessary to know the position and orientation of the acquisition system in the coordinate system defined by the target frame. This is also done using photogrammetric techniques. The acquisition system is placed inside the target frame and acquires another target-covered object. Several acquisitions of the target frame and object from various points of view are used to measure the respective position and orientation of the previously calibrated coordinate systems. This provides us with *T_C_F_,C_SI__*, the transformation between the acquisition system coordinate system and the frame coordinate system.

#### Orientation of the tracking cameras

To know the relative orientation of the tracking cameras, these cameras simultaneously acquire images of a calibration plate or a scale bar in several positions and orientations. For every camera *j*, we thus know *T*_*C*_0_,*C_Cj_*_.

The data processing is then performed in two steps:

1. The interior and exterior orientation of the tracking cameras and the images of the target frame from the tracking cameras are used to compute the position and orientation of the target frame for each acquisition. This provides us with *T_C_*_0_*_,CF_*. We refer to this as tracking; this is what the simulations describe.
2. Using the tracking results, the calibration between the tracking frame and the acquisition system, and the acquisition system calibration, we project the acquired data in a single coordinate system. We can thus calculate *A*∣*_C_*_0_, the coordinates of the surface points in the world system using:

(1) $${A|}_{C_{0}}=T_{C_{0},C_{F}}\cdot T_{C_{F},C_{Si}}\cdot {A|}_{C_{Si}}$$

This is what we call the registration.

### 2.2. Materials Used

In this section we describe the materials used during the experiments. We differentiate the acquisition systems that we used because they correspond to our acquisition problem, the additional material necessary to perform the optical tracking and the software used to process the data and perform the registration.

#### 2.2.1. Acquisition Systems

The multispectral acquisitions are performed with a commercial camera from FluxData (the FD-1665-MS [31]). This camera is based on a 3 CCD system that provides simultaneous data for each spectral band. A few characteristics of this camera are given in Table 1. It can acquire 7 spectral bands, six in the visible spectrum and one in the near infrared. This number of channels has been shown to be sufficient for pigment characterization [32]. Accurate spectral calibration and a neural network algorithm are able to provide us with a reflectance spectra for each pixel [33]. The big sensor and pixel size will allow us to precisely register the acquired data. This is a light and compact multispectral camera that is adapted for the in situ study of cultural heritage. For these acquisitions the camera was used with a LED ring light.

The 3D surface digitization is carried out by a commercial fringe projection system, the Atos III, manufactured by Gom [34]. The Atos is composed of two 4 Mpx cameras and a projector. Different acquisition configurations can be built by varying the position, orientation and lens of the cameras and projector. The output resolution is proportional to the dimensions of the field of view.

#### 2.2.2. Tracking Material

Material characteristics such as the tracking cameras' resolution and focal length as well as the target frame characteristics (dimensions and number of targets) were chosen through several rounds of simulations.

**Figure 2.**
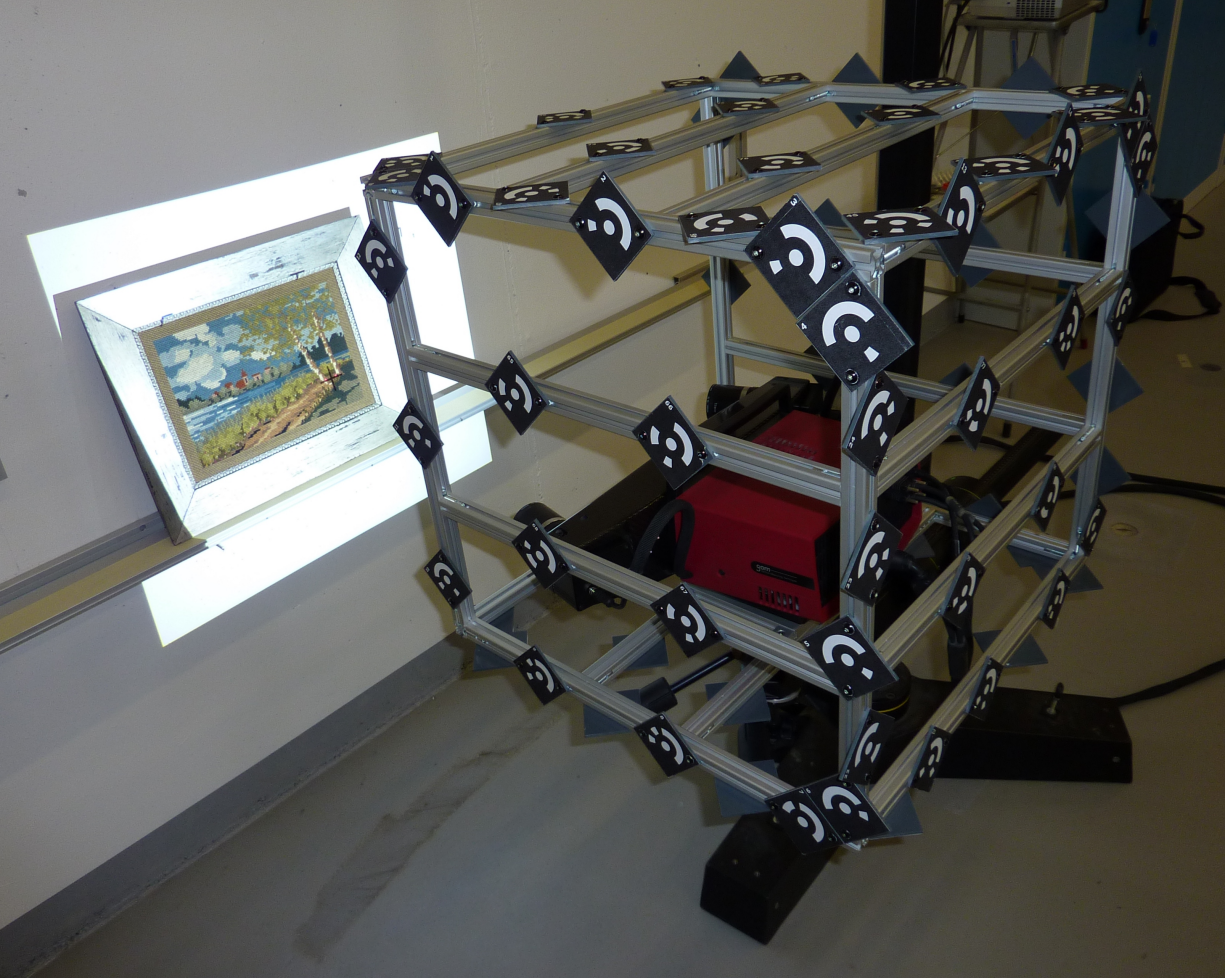
Gom Atos III in the target frame acquiring the cross-stitch canvas.

The tracking cameras are 5 Mpx grayscale cameras AVT Stingray F-504B. They are used with 8 mm lens from Pentax. Short focal lengths are necessary to provide a wide field of view for the tracking. The target frame is a cube of side 500 mm, covered with 80 targets (see Figure 2). It is made of aluminum profiles, which allow an easy and rapid prototyping. A hexagonal plate is fixed to the bottom of the frame to securely fix it to a tripod. A plate holder inside the cube is used to fix the acquisition systems to the frame. Additional materials used for the calibrations include an extra digital camera (we use a Nikon D300 camera with an external flash), scale bars and calibrated target plates.

#### 2.2.3. Processing Software

The processing of the calibration and acquisition photogrammetric bundles relies on two pieces of software. We use Gom's photogrammetric bundle adjustment software TriTop [35] to recognize the targets and perform an initial bundle adjustment. We thus estimate the cameras' internal and external orientation, the coordinates of the targets in a world system and the coordinates of the targets in the images.

The output is then processed by a lab-developed software based on the AxOri library [36], i3mainzAxOri. We now perform the bundle adjustment with greater flexibility in differentiating the unknowns from the input parameters. For example, we can estimate the target coordinates or the object position by assuming the tracking cameras' interior and exterior orientation are known. It is also possible to assess the exterior orientation of the tracking cameras assuming the interior orientation and the object coordinates are accurate. If we assume the tracking camera exterior orientation and the object coordinates are known, then we can determine the tracking cameras interior orientation. The bundle adjustment results also provide us with an internal accuracy measure of the parameters calculated. These are the accuracy values presented further on in this article, given at 2*σ*.

### 2.3. Configuration Overview

In addition to helping us select the tracking material, simulations enabled us to evaluate the achievable tracking accuracy in three configurations, chosen to represent a variety of cultural heritage objects. When the acquisition systems are fixed to this target frame and survey an area of 400 mm × 700 mm, the simulations [30] show that we can achieve a spatial tracking accuracy of 0.020 mm and an angular accuracy of 0.100 mrad using four tracking cameras. Equivalent tracking accuracy can be obtained using six cameras when the acquisition systems digitize an area of 2,000 mm × 1,500 mm, such as a wall-painting. If the object under study is a 1,000 mm high statue with a 300 mm radius then the target frame can be tracked with comparable spatial accuracy and an angular accuracy of 0.122 mrad. This, however, requires eight tracking cameras and would be harder to achieve in practice.

Simulations were run in a configuration that corresponds to a real case study: the digitization of the surface of a polychrome sarcophagus from the 3rd century. In this case study the area of interest measures 400 mm × 700 mm. The first experimental results presented here correspond to a reduced version of the initial configuration in which we digitize a framed cross-stitch canvas. The frame measures 450 mm × 360 mm while the cross-stitch covers an area of 320 mm × 230 mm. This is a good test object since it contains both spatial surface variations in the surface of the frame and in the stitches, as well as reflectance variations in the color of the thread. The frame sits on a small metallic shelf, and the four tracking cameras are positioned to observe the acquisition systems while they digitize the surface, as shown Figure 1.

Our goal is to register the data with an accuracy twice as good as the acquisition system resolution. This corresponds to a different target accuracy depending on the acquisition distance. We use the Gom Atos III in the configuration that provides a 500 mm × 500 mm field of view. This entails a 0.24 mm resolution and a fixed distance of 760 mm between the acquisition system and the object under study. In the case of the FluxData camera, we assume this distance to be 500 mm. This corresponds to an image of approximately 180 mm × 130 mm.

The resulting accuracy goal is given in Table 2. Spatially, the most restrictive target value is0.099 mm and angularly 0.158 mrad. The simulations have taught us that it is this angular accuracy that will be the hardest to reach.

## 3. Results and Discussion

The bulk of the calibrations were performed on the same day as the acquisitions. Only the camera calibrations were done at different moments: the tracking cameras were calibrated a few days earlier, while the multispectral camera was calibrated on the following day. The performed acquisitions and calibrations are listed in Table 3. This section first presents the results of the individual calibrations. We then evaluate the accuracy with which we track the target frame. Finally, we present the 3D registration results, followed by the integration of the 3D and multispectral datasets.

### 3.1. Individual Calibrations

The accuracy of the individual calibrations is given in Table 4. When available, the equivalent values from the simulations [30] are given for comparison.

#### Sensor calibrations

To determine the interior orientation of the tracking cameras, we acquire a calibration plate in approximately eighty positions. The resulting calibration is more accurate that the values used for the simulations. It would thus be theoretically possible to calibrate the cameras with fewer acquisitions; however we welcome this improved calibration.

A smaller calibration plate is used to independently calibrate each of the three CCDs of the multispectral camera. The calibration is not as accurate as the one performed with the tracking cameras. This is partially due to the reduced depth of focus of the multispectral camera, which limits the volume in which we can clearly identify the targets from the calibration plate.

The Gom Atos III has a specific calibration procedure similar to that described above.

#### Calibration of the target frame

The target frame is calibrated with over a hundred images. Each target is present in an average of over a third of the total images and the resulting accuracy beats the accuracy expected from the simulations.

#### Orientation of the tracking cameras

We use one camera coordinate system as the world coordinate system (*C*_0_ = *C_C_*_1_). The position and orientation of the other three tracking cameras are calculated in this system. Using approximately ninety images the spatial accuracy obtained is only slightly worse than the best expected results and better than the expected results angularly.

#### Orientation between the target frame and the acquisition systems

This orientation is calculated using three different relative positions of the target frame and calibration object. The discrepancy between the small field of view of the multispectral camera and the large tracking frame are the cause of the disappointing accuracy of the orientation of the tracking frame in the system defined by the multispectral camera. This orientation can be improved if the multispectral camera observes a greater number of targets: here each image could only see four or five targets of the calibration object. In a posterior measure we were able to attain an accuracy of 0.13 mm spatially and 0.43 mrad angularly when the multispectral camera observed seven to twelve targets.

### 3.2. Tracking

**Figure 3.**
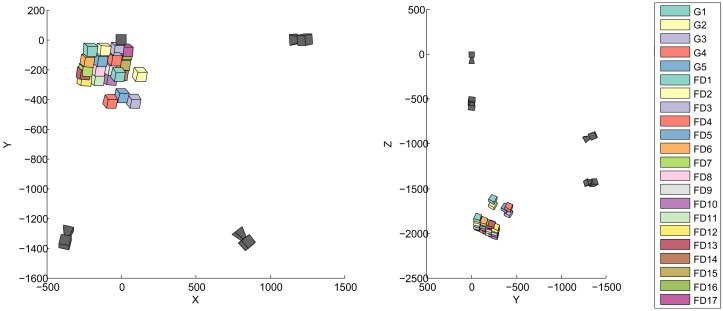
Relative position of the tracking cameras (dark gray) and target frame for all the acquisition positions.

The target frame is tracked in 23 positions: 5 when the Gom Atos III digitizes the scene (G1 to G5) and 17 for the FluxData multispectral acquisitions (positions FD1 to FD17). The relative position of the target frame and the cameras is illustrated in Figure 3.

**Figure 4.**
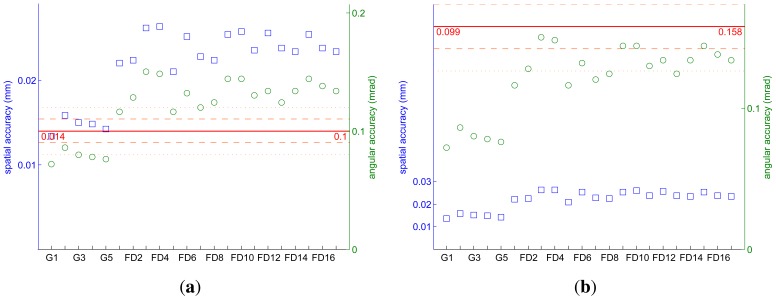
Spatial (blue squares) and angular (green circles) tracking accuracy compared with the best-case scenario simulation results (**a**) and to the tracking goal (**b**).

The spatial and angular tracking accuracy of the frame tracking for each position is given in Figure 4. The achieved tracking accuracy is compared with the simulation results in the best-case scenario and to the accuracy goal.

We only reach the simulation results for the angular accuracy when the Gom is in the target frame. The spatial accuracy is also much better for these five positions, rarely exceeding 10 % more than the spatial accuracy reached during the simulations. As shown in Figure 3, the target frame is closer to the cameras during the Gom acquisitions. These positions closely resemble those used for the simulations. It is thus not surprising that the tracking accuracy better corresponds to the simulation results.

If we compare the achieved accuracy to the tracking goal we notice that the tracking accuracy is always better than our goal. As expected from the simulations, we have no difficulty reaching our target spatial accuracy. Our worst spatial value (0.026 mm for FD3) is more than three times better than the spatial accuracy goal of 0.099 mm.

The least well tracked positions are 3, 4, 9, 10, and 15 of the FluxData multispectral cameras. These first four positions are those of the third column from the left. For these positions we have a perspective view of all targets on the left and right side of the frame. Even though there are a comparable number of targets detected by each camera compared with the other positions, since these targets are not well defined, the ensuing tracking is less accurate.

The tracking results could be improved by re-designing the tracking frame: If this frame were spherical instead of cubic, the tracking accuracy would not depend on its orientation with respect to the tracking cameras. Also, the current cube design is such that the targets hide one another from certain points of view. This could be solved if the targets did not stick out of the tracking frame. Finally, aluminum is convenient for rapid prototyping but a carbon tracking frame would be more stable and less sensitive to temperature changes that can occur over the course of a day.

### 3.3. 3D Registration

The frame of the cross-stitch canvas is difficult to digitize with the Gom Atos III, due to the shiny paint that covers it. The intricate decorations of the interior of the frame are particularly challenging to digitize and were only partially acquired.

**Figure 5.**
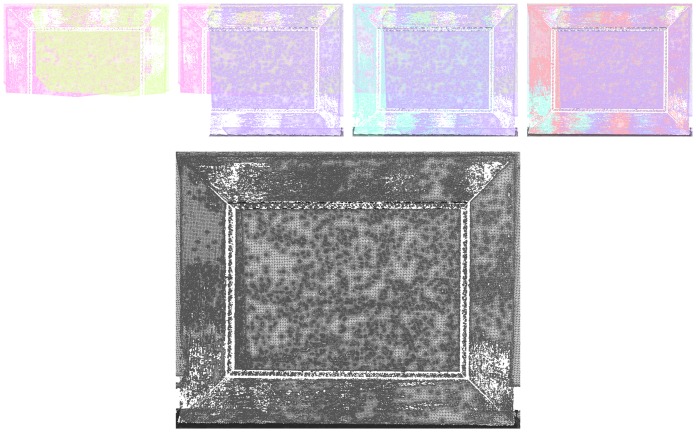
3D registration. First row: successive projection of each mesh. Bottom image: all meshes.

Figure 5 shows the registration of the five meshes as well as the final model. There are some holes remaining in this final model, particularly in the area representing the frame, but the cross-stitch canvas is almost fully acquired. There are no visible discontinuities in the 3D model, although these would be easily visible on the exterior and interior edges of the frame.

The theoretical accuracy of the final registration is the sum of the tracking accuracy and the target frame to acquisition system orientation. In the case of the 3D data, the theoretical spatial accuracy is thus the sum of the spatial accuracy of the least well tracked position of the Gom Atos III, position G2, (0.016 mm) and of the spatial accuracy of the target frame to Gom Atos III orientation (0.029 mm). This theoretical spatial accuracy of 0.045 mm is well below our target accuracy of 0.09 mm. The theoretical angular accuracy is 0.158 mrad (0.0860 mrad, angular accuracy of G2, plus 0.072 mrad, spatial accuracy of the target frame to Gom Atos III orientation). This is exactly our target angular accuracy. We reach our target 3D registration accuracy somewhat narrowly and based on the assumption that the calibration of the 3D digitization system introduces negligible errors.

The advantage of this method is that the registration inaccuracy is independent of the content of the data: if the acquired data represented a smooth plane, it would be registered with the same accuracy. This is not the case with the Iterative Closest Point algorithm (ICP) [37,38], which generally fares badly with smooth and planar data. Furthermore, the deviation errors produced using ICP would accumulate across the views. Also, ICP requires a 30 % to 40 % overlap between adjacent meshes. Though we have such overlap in this data, it does not influence the success of the registration.

### 3.4. 3D / Multispectral Registration

**Figure 6.**
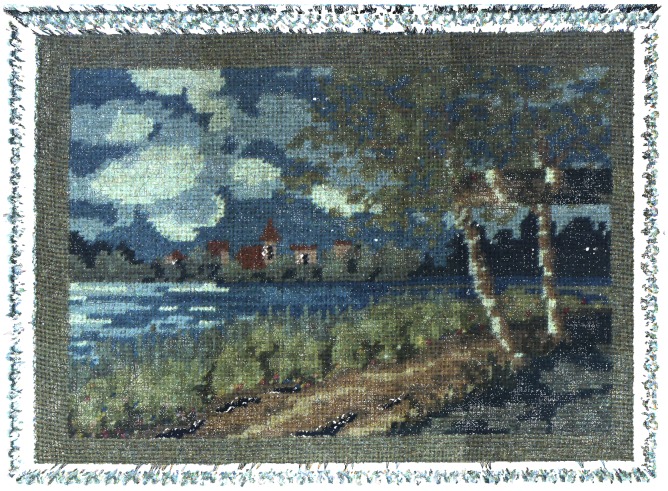
Multispectral/3D registration.

Figure 6 shows the full registration of multispectral acquisitions on the 3D model. For these acquisitions we only used the six channels in the visible range from the FluxData multispectral camera. For visualization purposes, we combine the channels two by two to create a color image. The seventeen images are successively projected on the 3D model in the order they were acquired. Areas captured several times are simply hidden by the following acquisitions.

**Figure 7.**
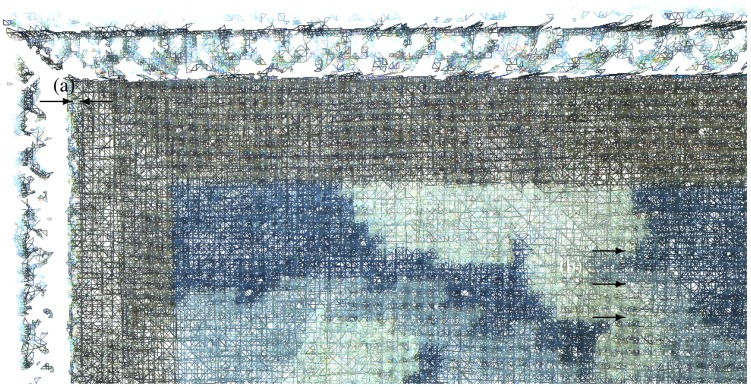
Closeup of the multispectral / 3D registration. (**a**) shows the inaccuracy of the 3D to multispectral registration while (**b**) highlights the seamless multispectral registration.

Visual examination of the 3D model presents a horizontal inaccuracy. Manually selecting two points, the mesh shows this inaccuracy to be of approximately 0.8 mm between the multispectral and 3D data, highlighted in Figure 7. There is no noticeable registration inaccuracy in the vertical direction. The 3D model and the images are well aligned both along the edge of the cross-stitch and along the edge of the engraving.

The theoretical spatial accuracy is only 0.950 mm: 0.026 mm, the accuracy of the least well tracked position of the multispectral camera, position FD4, plus 0.924 mm, the spatial accuracy of the target frame to multispectral camera orientation. This inaccuracy is thus mostly due to the inaccuracy of the target frame to multispectral camera orientation. We observe the same problem on the theoretical angular accuracy; of the total 3.306 mrad, 3.156 mrad are due to the orientation of the target frame to the multispectral camera, while only 0.150 mrad come from the tracking accuracy (position FD3). This explains why we have a good multi-view registration of the multispectral data, even though the multimodal registration could be improved. The accuracy of the multispectral registration on the 3D model could be greatly improved with a better calibration between the multispectral camera and the target frame.

Another important factor for the accurate registration of the multispectral data on the 3D model is the accuracy of the camera interior orientation. The accuracy of the focal length ensures that the image is correctly scaled, it is also most important to compensate the distortion of the lens, though this is not a visible problem here. An inaccurate calibration of the principle point offset, however, could introduce the type of error visible here.

## 4. Conclusions and Perspectives

Using a photogrammetric setup based on four tracking cameras and a cubic target frame with a side length of 500 mm, we were able to successively track the position and orientation of a fringe projection digitization system and a multispectral camera with an accuracy better than 0.03 mm spatially and 0.150 mrad angularly. The tracking results are used to register five 3D meshes together and to project seventeen multispectral acquisitions. These first experimental results show that our tracking method is adapted not only for the registration of 3D datasets, but also for the integration of multispectral texture on 3D models.

The accuracy of the final registration relies on the success of a series of optical and geometrical calibrations. It is essential that the calibrated parameters are stable for the duration of the full acquisition process, typically from four to eight hours. The global setup of this technique is cumbersome and the registration of 3D and multispectral data requires up to seven calibrations. Though they are not difficult to perform, these photogrammetric calibrations are time consuming. Nevertheless, a well organized team of two can perform all the necessary calibrations and acquisitions in a single day (including the camera calibrations, though this was not the case here).

The use of photogrammetry offers a high flexibility: the setup can easily be adapted to the dimensions of the objects under study. For example, simulations have shown that an equivalent tracking accuracy can be obtained when using six cameras to survey an area of 2,000 mm × 1,500 mm.

All the necessary materials are transportable. The weight of the prototype target cube (currently 9.6 kg) and camera supports (30 kg) can be reduced by using lighter materials. In the current configuration, the weight of this material is comparable to that of a laser tracker (the Leica Absolute Tracker, for example, weighs 39 kg).

A big advantage of this technique is that it can be used with any optical acquisition system that the user may already possess. The additional cost is thus only that of the tracking frame, tracking cameras and lens, which in our case is under 15,000 €. This is much less than the cost of a laser tracker, though this amount does not include software costs. A broader use of the technique would require the development of an independent and integrated software tool for data processing and display.

The method is particularly cumbersome when digitizing highly three dimensional objects: eight tracking cameras may be necessary for a 1 m high statue. As the number of cameras increase, so does the number of calibrations necessary and the difficulty of maintaining a stable setup.

Compared with automatic alignment method, our system offers clear advantages when dealing with object with low spatial variability. Our system offers a solution to the multi-view and multimodal registration of smooth and featureless datasets. The registration accuracy is independent of the content of the acquired data, which means that the errors do not accumulate during the 3D registration of open surfaces, as would be the case using ICP, for example. These characteristics, including the fact that it is transportable, make our technique particularly suitable for the in situ study of cultural heritage objects.

This registration method can also be used in industrial settings. In such a fixed setup, the tracking accuracy can be increased. Also, the calibrations will only have to be performed from time to time, rather than once per object under study as in the case of the in situ study of cultural heritage. Furthermore, the technique works independently from the acquisition systems used, as long as they are based on optical sensors that can be characterized and calibrated. Data from thermal sensors or other optical sensors can provide complementary texture for the 3D model.

## Figures and Tables

**Table 1.** FluxData multispectral camera characteristics.

Characteristic	Value	Unit
Filtering technology	3 CCD	
Number of spectral bands	7	
Sensor size (W × H)	8.9 × 6.7	mm × mm
	694 × 494	pixels × pixels
Cell size	9.9	μm
Focal length	25	mm
Acquisition range	400–950	nm
External dimensions	92 × 112 × 187	mm × mm×mm
Weight	1.25	kg

**Table 2.** Tracking accuracy goal for each acquisition system. Most restrictive values are shown in boldface.

Acquisition system	Acquisition distance (mm)	Accuracy goal	
		spatial (mm)	angular (mrad)
FluxData multispectral camera	500	0.099	0.198
Gom Atos III digitization system	760	0.120	0.158

**Table 3.** List of calibrations and acquisitions performed.

Tracking cameras calibration
Multispectral camera calibration
Gom Atos III calibration

Target frame calibration
Tracking cameras orientation

Multispectral camera to target frame orientation
Multispectral acquisitions with simultaneous tracking

Gom Atos III to target frame orientation
Gom acquisitions with simultaneous tracking

**Table 4.** Accuracy of the individual calibrations compared with the simulation values.

Calibration	Measures	Simulations		Unit
		realistic	best	
Tracking cameras calibration	0.029	0.1	0.033	pixel
	
Multispectral camera calibration	0.035	—		pixel
	
Target frame calibration	0.011	0.0	50	mm

Tracking cameras orientation	0.011	0.03	0.01	mm
	0.014	0.04	0.02	mrad

Target frame to multispectral	0.924	—		mm
camera orientation	3.156	—		mrad
	
Target frame to Gom	0.029	—		mm
Atos III orientation	0.072	—		mrad
